# Identifying the potential causal role of insomnia symptoms on 11,409 health-related outcomes: a phenome-wide Mendelian randomisation analysis in UK Biobank

**DOI:** 10.1186/s12916-023-02832-8

**Published:** 2023-04-03

**Authors:** Mark J. Gibson, Deborah A. Lawlor, Louise A. C. Millard

**Affiliations:** 1 MRC Integrative Epidemiology Unit (IEU), University of Bristol, Bristol, UK; 2 School of Psychological Science, University of Bristol, Bristol, UK; 3 Department of Population Health Sciences, Bristol Medical School, University of Bristol, Bristol, UK

**Keywords:** Insomnia, Mendelian randomisation, MR-PheWAS, UK Biobank

## Abstract

**Background:**

Insomnia symptoms are widespread in the population and might have effects on many chronic conditions and their risk factors but previous research has focused on select hypothesised associations/effects rather than taking a systematic hypothesis-free approach across many health outcomes.

**Methods:**

We performed a Mendelian randomisation (MR) phenome-wide association study (PheWAS) in 336,975 unrelated white-British UK Biobank participants. Self-reported** i**nsomnia symptoms were instrumented by a genetic risk score (GRS) created from 129 single-nucleotide polymorphisms (SNPs). A total of 11,409 outcomes from UK Biobank were extracted and processed by an automated pipeline (PHESANT) for the MR-PheWAS. Potential causal effects (those passing a Bonferroni-corrected significance threshold) were followed up with two-sample MR in MR-Base, where possible.

**Results:**

Four hundred thirty-seven potential causal effects of insomnia symptoms were observed for a diverse range of outcomes, including anxiety, depression, pain, body composition, respiratory, musculoskeletal and cardiovascular traits. We were able to undertake two-sample MR for 71 of these 437 and found evidence of causal effects (with directionally concordant effect estimates across main and sensitivity analyses) for 30 of these. These included novel findings (by which we mean not extensively explored in conventional observational studies and not previously explored using MR based on a systematic search) of an adverse effect on risk of spondylosis (OR [95%CI] = 1.55 [1.33, 1.81]) and bronchitis (OR [95%CI] = 1.12 [1.03, 1.22]), among others.

**Conclusions:**

Insomnia symptoms potentially cause a wide range of adverse health-related outcomes and behaviours. This has implications for developing interventions to prevent and treat a number of diseases in order to reduce multimorbidity and associated polypharmacy.

**Supplementary Information:**

The online version contains supplementary material available at 10.1186/s12916-023-02832-8.

## Background

While there is still much debate over the exact purpose of sleep, it is clear that sleep is vital for healthy functioning and likely to be multifaceted. Experiments on rats have suggested that sleep is linked to antioxidative enzyme levels in the brain which regulate the levels of reactive oxygen species (by-products of the metabolization of oxygen which damage cells) [1]. It has also been proposed that sleep is vital for the consolidation of information, learning, and memory [2, 3]. Insomnia is defined as regular dissatisfaction with the quality or quantity of sleep for a prolonged period and includes difficulty initiating or maintaining sleep [4]. Evidence suggests that 6–7% of the European population have a diagnosis of insomnia, while 33–37% self-report having insomnia symptoms [5–7]. It is the second most prevalent mental health disorder (after anxiety disorder) and is more common in women and the elderly [6, 7]. Multimorbidity, defined as patients living with two or more chronic health conditions, is associated with polypharmacy, poor quality of life and premature mortality [8, 9]. It is increasingly recognised as a threat to global health and identifying potential causes of multimorbidity is a research priority [10].

Given the high prevalence of insomnia symptoms, and their potentially causal associations with many diseases (including increased risk of depression [11, 12], substance use [13, 14], autism spectrum disorder and bipolar disorder [15], dementia [16], high body mass index and diabetes [17, 18], hypertension [19], cardiovascular disease [20–22], pain [23] and inflammation [24]), insomnia symptoms could lead to multimorbidity. However, studies to date have largely been observational and may not reflect causal effects, and/or have focused on hypothesised selected outcomes, predominantly mental, neurocognitive and cardiometabolic outcomes, rather than systematically, using a hypothesis free approach, searching for potential causal effects across a wide range of health and disease outcomes. If insomnia symptoms are a cause of multimorbidity then insomnia treatments, such as cognitive behavioural therapy for Insomnia [25] recommended by UK National Institute for Health and Care Excellence [26], might be an effective means of reducing other diseases and multimorbidity, in those with insomnia.

Mendelian randomisation (MR) is a method used for testing causal relationships that generally uses genetic variants that are robustly associated with the exposure of interest as instrumental variables (IV) [27]. MR is typically less prone to confounding of the exposure-outcome association and reverse causation than conventional observational epidemiology; as genetic variation is determined at conception, it cannot be altered by disease status [28]. However, it has other potential sources of bias, in particular those due to weak instruments, confounding of the instrument-outcome association and horizontal pleiotropy [29] (the core assumptions of MR have been previously reported in detail [30]). A MR-phenome-wide association study (MR-PheWAS) is a hypothesis-free approach that tests for causal effects of a trait of interest [31] on many phenotypes [32]. To our knowledge, only one previous study has undertaken an MR-PheWAS of insomnia symptoms [33]. In that study, the automated tool PhenoScanner [34] was used to explore causal effects of maternal insomnia symptoms on 17,503 outcomes. It identified 2844 potential causal effects (*p*-value < 0.05) including on adiposity, mental health, musculoskeletal, respiratory/allergic and reproductive phenotypes. However, that MR-PheWAS was part of an illustrative example in a methodological paper focused on addressing one of the MR assumptions, and none of the potential causal effects were explored further with replication or sensitivity analyses. The aim of this study is to explore the causal effects of insomnia symptoms on a wide-range of disease and health-related traits. We followed the STROBE-MR reporting guidelines when writing this paper [35] and this study was not pre-registered.

## Methods

### Study population

We used data from UK Biobank, a large prospective cohort study (dataset ID 43017 of UK Biobank application 16729, phenotypic data extracted on 24/02/2021). UK Biobank recruited 503,325 adults aged from 37 to 73 years. They were recruited between 2006 and 2007 and attended one of the 22 test centres across the UK. Of the 503,325 participants, genetic data (see Additional file 1: Text S1) was successfully obtained for 487,406 participants [36]. Participants were then excluded from this sample if they did not meet the genetic quality control [37], they were not of white-British ancestry, they were not part of the maximal subset of individuals not related to any other individual to the third degree or higher or they had since withdrawn their consent (as of 09/08/2021). The remaining 336,975 participants were included in the MR-PheWAS (See Additional file 1: Fig. S1 for a flow diagram).

### Genetic risk score

We generated a weighted genetic risk score (GRS) using 129 independent single-nucleotide polymorphisms (SNPs) previously identified [18] to associate with self-reported insomnia symptoms (answering yes to any of eight questions about insomnia diagnosis, symptoms or treatment versus answering no to all these question plus three more questions about diagnosis and treatment of collections of diseases which include insomnia—see Additional file 1: Text S2) at GWAS significance (with *p* < 5 × 10^−8^) in 23andMe, Inc. (Additional file 2: Table S1). These data were requested from 23andMe as they were not provided in the original GWAS paper. SNPs were weighted by their per-allele association with insomnia symptoms in the original GWAS. We used a linkage disequilibrium (LD) threshold of *R*^2^ > 0.001 to clump the GWAS significant SNPs into independent SNPs. LD was calculated in the 1000 Genomes European data [38], and the TwoSampleMR (MR-base) R package v0.5.6 [39] was used to clump GWAS significant SNPs into independent SNPs. One SNP (rs28458909) was not available in UK Biobank and thus was replaced by a proxy (rs28780988) that was in close LD (*R*^2^ = 1). All palindromic SNPs had an effect allele frequency falling below 0.49 or above 0.51 in UK Biobank and 23andMe and therefore could be harmonised.

As the SNPs used to construct the GRS are not replicated, there is a higher chance that spurious SNPs could have been falsely detected. We created two sensitivity analysis GRS which used SNPs which were replicated in a meta-analysis of 23andMe and UK Biobank. These analyses are only sensitivity analyses as they are at risk of overfitting due to UK Biobank being used to identify SNPs (see Additional file 1: Text S3 and Additional file 2: Table S2).

### Outcomes

A total of 11,409 outcome variables were derived and analysed using PHESANT [40]. Outcomes included those obtained from responses to baseline and follow-up questionnaires, baseline assessments such as weight, height, blood pressure and bone density measurements, follow-up assessments such as accelerometer measurements and a range of different scans (including brain and cardiac scans), biomarker measures from blood or urine samples and outcomes from linkage to primary and secondary care, and the national cancer and death registers. In order to summarise our overall findings from the MR-PheWAS, outcomes were assigned to categories and subcategories based on their UK Biobank category (e.g. Online follow-up > Mental health > Anxiety). Measurements that were not health-related outcomes were assigned to the Auxiliary Variables category. These included outcomes such as hospital administration records and procedural metrics. Individual sleep variables from the mental health and physical health categories were then reassigned to a sleep category and medication variables in the physical health category that were for mental disorders were reassigned to the mental health category. We then manually assigned outcomes in these two categories to subcategories.

### MR-PheWAS analysis

The PHESANT package (v1.0) was used for the MR-PheWAS. We adjusted for age at assessment, sex and the top 10 genetic principal components to control for populations stratification [41]. A complete case analysis was undertaken by PHESANT meaning participant numbers differ between outcomes and we chose to exclude outcomes with less than 100 cases. PHESANT derives outcomes from the UK Biobank data and defines whether they are continuous, binary, ordered categorical or unordered categorical and tests the association with a trait of interest, in our case the insomnia symptoms GRS, using linear (using inverse normal rank transformed data to ensure a normal distribution), logistic, ordered logistic, and multinomial logistic regression, respectively. The results are presented as difference in mean standard deviation (SD) of inverse rank normal transformed continuous outcomes and odds ratio (OR) for categorical outcomes, per 1 SD increase in the weighted GRS. We defined *potential causal effects* as any insomnia symptoms GRS-outcome association that passed the Bonferroni-corrected significance threshold of 4.38 × 10^−6^ (0.05/11,409) in the MR-PheWAS. The less conservative false discovery rate correction was also calculated and reported but was not used to identify potential causal effects for follow-up.

### Follow-up two-sample MR

We undertook follow-up analyses using two-sample MR for all outcomes for which the association with the GRS was identified as a potential causal effect of insomnia symptoms and an appropriate GWAS could be found. The purpose of this was to confirm the reliability of the potential causal effects identified in the MR-PheWAS and to provide a causal estimate. The TwoSampleMR package (MR-base) v0.5.6 [39] was used to conduct the follow-up. It was decided *a priori* that outcomes included in the auxiliary variables or sleep categories would not be followed up. We conducted an automated search for relevant GWAS using pre-specified search terms for each outcome and a predetermined workflow to select the most appropriate GWAS for each outcome. First, we conducted an automated search for relevant GWAS using pre-specified search terms for each outcome. The search automatically excluded GWAS that included solely UK Biobank data, included non-European populations or stratified by sex, based on the meta-data included in the MR-Base database. Of the remaining GWAS, we excluded those that did not match a follow-up outcome on manual inspection, those for which the origins of the data used could not be determined and those that used UK Biobank or 23andMe data. If the only GWAS available for a particular outcome included UK Biobank or 23andMe data (but did not only include UK Biobank or 23andMe data), we undertook follow-up in those GWAS and report the extent of overlap between the two samples. Of the remaining GWAS, we then chose the most suitable for a given trait. This was either the most suitable match in terms of the trait used in that GWAS or where multiple GWAS had suitable traits, we chose the one with the larger sample size. All GWAS from FinnGen were then updated to the newest version when the fifth release was added to the MR-Base database.

The two-sample MR analysis used the same 129 SNPs and SNP-insomnia symptoms associations used by the MR-PheWAS GRS [18], and the SNP-outcome associations were extracted from the GWAS for each outcome. We used the TwoSampleMR (MR-base) package for the two-sample MR analyses, which has a built-in function for harmonising SNPs between the SNP-exposure and SNP-outcome summary results (in this study so that results reflect the effects of having symptoms on outcomes for each SNP). By default, SNPs are excluded if harmonisation is not possible (e.g. if a suitable proxy cannot be found for missing SNPs or if SNPs were palindromic with allele frequencies near to 0.5). We used the inverse-variance weighted (IVW) method for our main two-sample MR analyses [42] and weighted median regression MR [43] and MR-Egger [44] as sensitivity analyses to explore potential bias due to unbalanced horizontal pleiotropy. We did not correct for multiple testing as these analyses only followed up results which had past the very conservative Bonferroni-corrected threshold used in the MR-PheWAS. All code can be found at https://github.com/MRCIEU/PHESANT-MR-PheWAS-Insomnia v1.1.

### Systematic search of previous literature

At the suggestion of a peer reviewer, we undertook a systematic search to identify published MR studies of the effect of insomnia on health outcomes. This was used to explore the extent to which the MR-PheWAS identified novel findings that have not been previously studied with MR. We searched Embase and Web of Science on 8/12/2022 for articles containing “Insomnia” AND (“Mendelian randomisation” OR “Mendelian randomization”) in any field. We excluded articles which were not fully peer-reviewed original research articles or were not investigating the causal effect of insomnia on an outcome through MR. We then extracted information on the relevant analyses from each article and whether they found evidence of a causal effect.

## Results

The study population had a mean age of 57 years, 54% were female and 32% were educated to degree level (Table 1). Self-reported insomnia symptoms were common, with 48% reporting these sometimes and 28% usually.

**Table 1:** Baseline characteristics for the white-British UK Biobank sample of 336,975 individuals included in the MR-PheWAS

	Mean (SD) or *N* (%)^a^
Age at assessment centre (years)	57 (8)
Townsend area deprivation score	− 1.58 (2.93)
Sex	336,975 (100%)
Male	155,702 (46%)
Female	181,269 (54%)
Insomnia	336,744 (99.9%))
Usually	95,380 (28%)
Sometimes	160,877 (48%)
Never/Rarely	80,483 (24%)
Education	333,846 (99%)
College or university degree	106,741 (32%)
A levels/AS levels or equivalent	38,439 (11%)
O levels/GCSEs or equivalent	74,089 (22%)
CSEs or equivalent	18,114 (5%)
NVQ/HND/HNC or equivalent	22,097 (7%)
Other professional qualifications (e.g. nursing or teaching)	17,284 (5%)
None	57,078 (17%)

*A level* advanced level, *AS level* advanced subsidiary level, *CSE* certificate of secondary education, *GCSE* General Certificate of Secondary Education, *HND* Higher National Diploma, *HNC* Higher National Certificate, *NVQ* National Vocational Qualification, *SD* standard deviation

^a^Mean (SD) for continuous variables and number and percentage for categorical variables

### MR-PheWAS

The insomnia symptoms GRS was associated with an increased risk of insomnia symptoms in UK Biobank: OR of self-report of usually versus never/rarely/sometimes having trouble falling or staying asleep = 1.08 [95% Confidence Interval (CI): 1.07, 1.09] per one standard deviation higher GRS (*p* = 3.59 × 10^−84^, McFadden’s pseudo *R*^2^ = 0.01). See Additional file 1: Fig. S2 for the association of each SNP with insomnia symptoms.

**Fig. 1:**
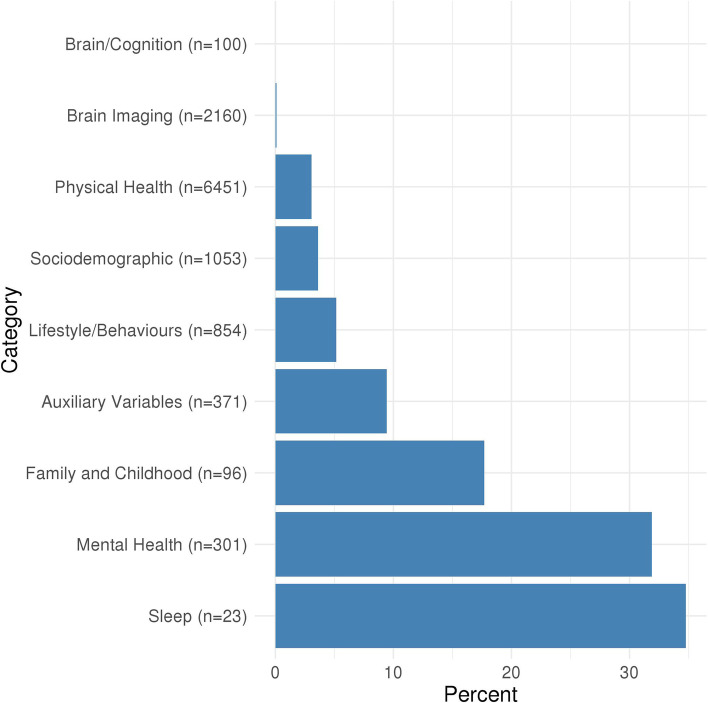
Proportion of potential causal effects of insomnia on outcomes within different categories. *n* is the total number of outcomes in the category. Additional file 2: Table S3 gives the category for each outcome. Results shown in this figure are also provided in Additional file 2: Table S4

**Fig. 2:**
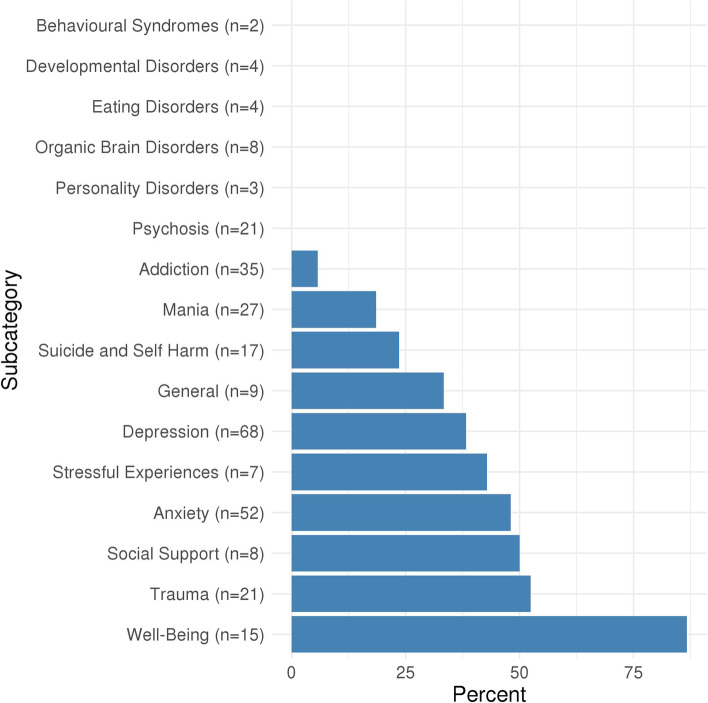
Proportion of potential causal effects of insomnia on outcomes within different mental health subcategories. *n* is the total number of outcomes in the category. Additional file 2: Table S3 gives the subcategory for each outcome. Results shown in this figure are also provided in Additional file 2: Table S5

**Fig. 3:**
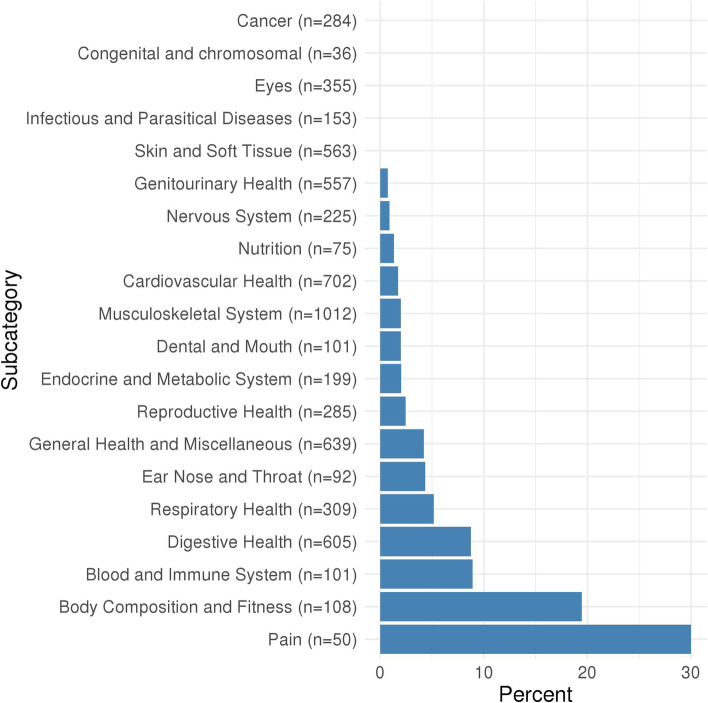
Proportion of potential causal effects of insomnia on outcomes within different physical health subcategories. *n* is the total number of outcomes in the category. Additional file 2: Table S3 gives the subcategory for each outcome. Results shown in this figure are also provided in Additional file 2: Table S5

Of the 11,409 associations included in the MR-PheWAS, 437 were identified as potential causal effects (Additional file 2: Table S3). These included anxiety, stress, depression, mania, addiction, pain, body composition, immune, respiratory, endocrine, dental, musculoskeletal, cardiovascular and reproductive traits, as well as socioeconomic and behavioural traits. Figure 1 shows the proportion of potential causal effects of insomnia symptoms by broad categories of outcomes. For associations between insomnia symptoms and mental health-related outcomes, 96 of 301 (32%) were identified as potential causal effects. There were higher proportions of these in 10 out of 17 of the mental health subcategories (Fig. 2), including depression (38%), anxiety (48%), general (33%), well-being (87%), suicide and self-harm (24%) and mania (19%). Of the physical health category, 197 out of 6451 (3%) associations with the insomnia symptoms GRS were identified as potential causal effects. Higher proportions of potential causal effects (Fig. 3) were seen for the pain (30%) and body composition (19%) subcategories. For the family and childhood category, 17 out of 96 (18%) associations were identified as potential causal effects. This category included some outcomes that could not be plausibly affected by adult insomnia and might reflect shared family (inherited) predisposition to insomnia and its potential causal effects on fertility and health-related outcomes across family members. For the lifestyle/behaviours category, 44 out of 854 outcomes (5%) were identified as potential causal effects, while for the sociodemographic category 38 out of 1053 (4%) were. There were 2 of 2160 (0.1%) outcomes identified as potential causal effects from the brain imaging category. Alternatively, the brain/cognition category had no potential causal effects. Full details of the numbers in each category/subcategory and the numbers and percentages of outcomes in those categories that are potentially influenced by insomnia symptoms are provided in Additional file 2: Tables S4 and S5. For the results of the sensitivity analyses, see Additional file 1: Text S4, Figs. S3-S4 and Additional file 2: Table S3.

### Follow-up two-sample MR

**Fig. 4:**
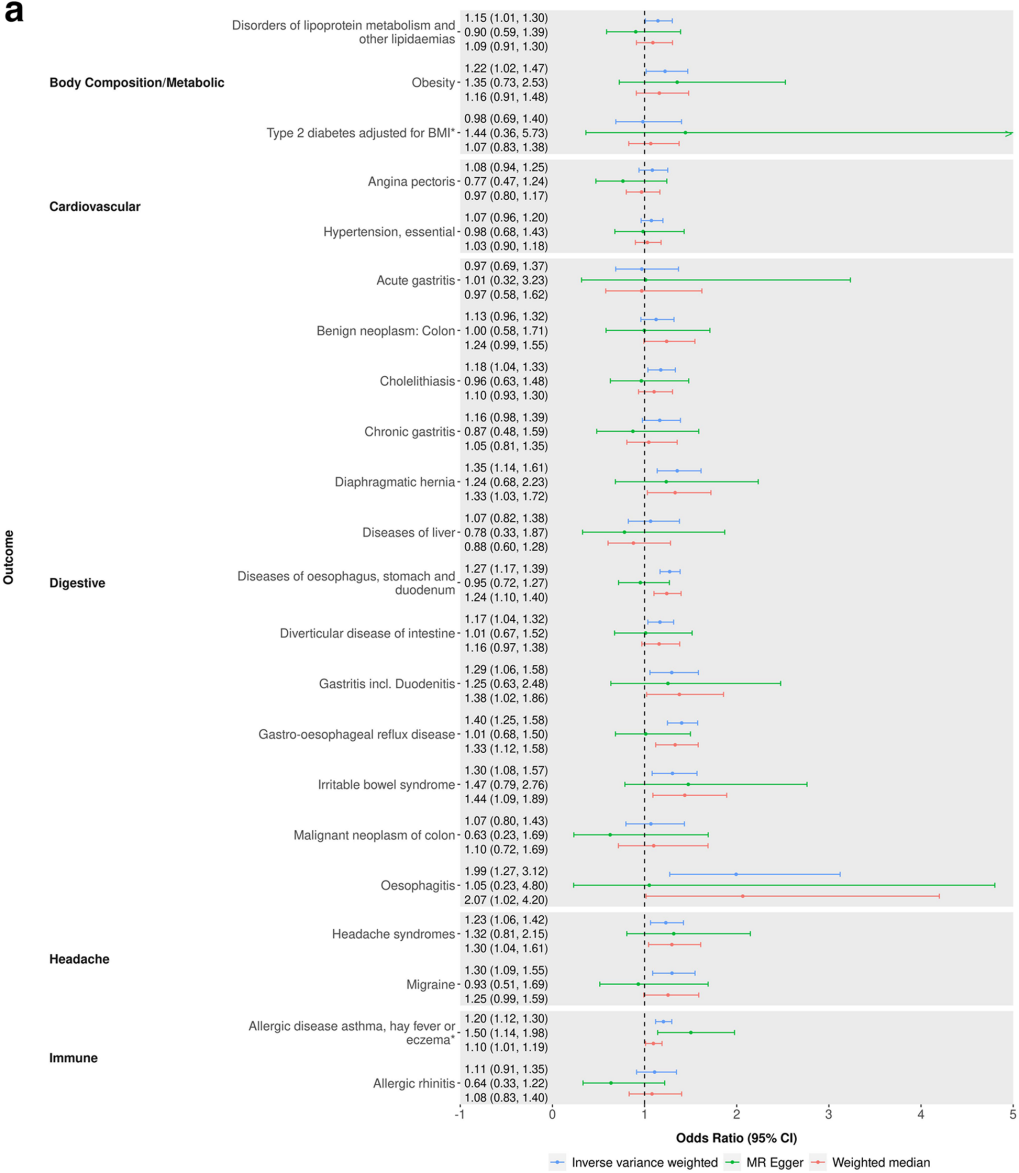

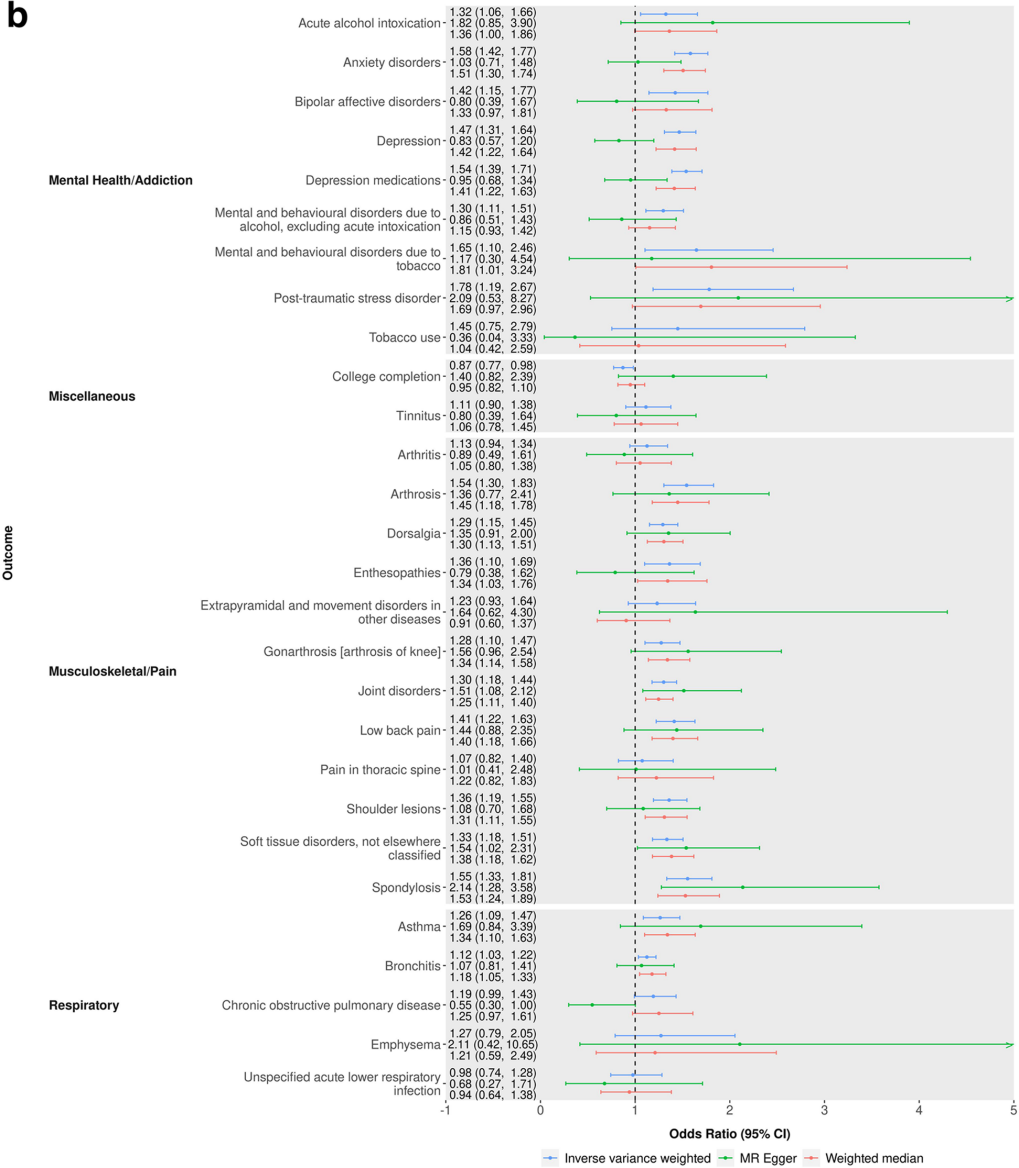
**a, b** Two-sample MR results of the effect (odds ratio), comparing genetically predicted self-reported insomnia cases versus non-cases for binary outcomes. *GWAS has overlap with UK Biobank or 23andMe

**Fig. 5:**
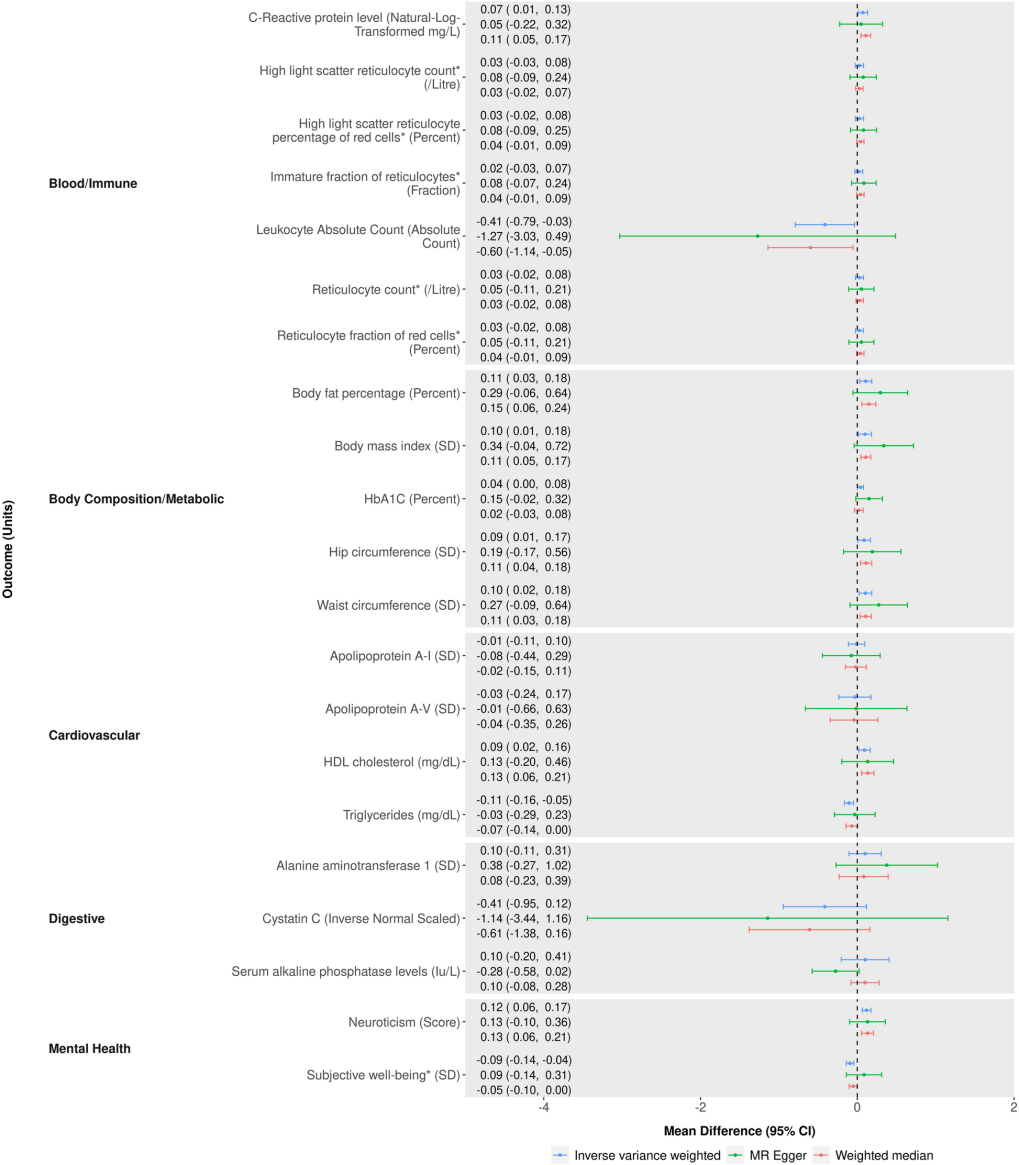
Two-sample MR results of the effect (mean difference), comparing genetically predicted self-reported insomnia cases versus non-cases, for continuous outcomes. *GWAS has overlap with UK Biobank or 23andMe

Of the 437 potential causal effects identified in the MR-PheWAS, we identified 71 with a relevant GWAS in MR-Base [45–132], and hence eligible for follow-up (see Additional file 1: Fig. S5 and Additional file 2: Tables S6-S8). Of these, 45 outcomes showed clear evidence of an effect of being a self-reported insomnia symptoms case versus not in the IVW MR analyses, having 95% CIs which excluded the null (Figs. 4a, b and 5 and Additional file 2: Tables S9-S10). Three of these estimates (HDL cholesterol, triglycerides and absolute leukocyte count) contradicted the direction of the MR-PheWAS estimate. Of the 42 remaining, 30 (7 continuous and 23 binary) of these had effect estimates in the same direction across all main and sensitivity two-sample MR analyses although with CIs often including the null. These 30 outcomes include a range of categories: substance use and mental health-related outcomes such as acute alcohol intoxication, mental and behavioural disorders due to tobacco, neuroticism, anxiety disorder and post-traumatic stress disorder; body composition outcomes such as obesity, body fat percentage, body mass index, hip circumference and waist circumference; musculoskeletal outcomes such as low back pain, gonarthrosis, unspecified arthrosis, unspecified joint disorders, shoulder lesions, unspecified soft tissue disorders, spondylosis and dorsalgia; digestive health-related outcomes such as irritable bowel syndrome, diverticular disease of intestine, unspecified gastritis (including duodenitis), gastro-oesophageal reflux disease, diaphragmatic hernia and oesophagitis; allergy or respiratory outcomes such as allergic disease (asthma, hay fever or eczema), asthma and bronchitis; and outcomes which were not related to others in the set such as unspecified headache syndromes, C-reactive protein level and HbA1c. Cochran’s Q showed evidence of between SNP heterogeneity (*p* < 0.05) in both the IVW and MR-Egger analyses for 16 of these 30 outcomes: Anxiety, asthma, obesity, body mass index, body fat percentage, hip circumference, waist circumference, C-reactive protein level, unspecified arthrosis, unspecified joint disorders, unspecified soft tissue disorders, shoulder lesions, low back pain, gonarthrosis, dorsalgia and allergic disease. Only anxiety disorders showed evidence of unbalanced horizontal pleiotropy in the MR-egger intercept, implying that heterogeneity in most SNP estimates is due to either balanced pleiotropy or different causal biological mechanisms of the SNP on insomnia symptoms.

### Systematic search of previous literature

After deduplication, abstract review and full-text review, 81 articles exploring the effect of insomnia on a health outcome via MR were identified in the systematic search (see Additional file 1: Fig. S6). Article information and a summary of the findings for each article included can be seen in Additional file 2: Table S11 (while information for articles excluded at full-text screening with the reason for exclusion can be seen in Additional file 2: Table S12). These articles showed evidence that insomnia may have causal effects on anxiety, neuroticism, posttraumatic stress disorder, subjective well-being, depressive symptoms, major mood disorder, a range of cardiovascular outcomes (including coronary heart disease, angina pectoris and hypertension), type 2 diabetes mellitus, cholesterol levels, body mass, osteoarthritis, rheumatoid arthritis, pain, migraine, gastro-oesophageal reflux disease, irritable bowel syndrome, miscarriage, allergic disease, asthma, smoking and alcohol use, among others. Of the 30 directionally consistent findings across the MR-PheWAS, two-sample follow-up MR and two-sample sensitivity analyses (for which the 95% CI excluded the null in the MR-PheWAS and the IVW two-sample follow-up), only spondylosis, unspecified joint disorders, shoulder lesions, unspecified soft-tissue disorders, gastritis (including duodenitis), oesophagitis, diverticular disease of intestine, diaphragmatic hernia, bronchitis, unspecified headache syndromes and C-reactive protein levels were not supported by previous MR literature (i.e. no clear evidence of a concordant evidence in the previous literature). While the systematic search identified no papers investigating the effects of insomnia on acute alcohol intoxication, mood and behavioural disorders due to tobacco, certain body composition outcomes and gonarthrosis (arthrosis of the knee) specifically, there was evidence for closely related and overlapping outcomes in the previous literature.

## Discussion

In this study, we conducted an MR-PheWAS of insomnia symptoms using 11,409 outcome variables. Of these GRS-outcome associations, 437 met our criteria for being potential causal effects, of which 71 were possible to follow-up using two-sample MR. Follow-up analyses showed consistent evidence of an adverse causal effect of insomnia symptoms on 30 outcomes including those related to anxiety disorders, respiratory disorders, musculoskeletal disorders, disorders of the digestive system and body composition measurements. A number of these had not previously been investigated using MR. These included respiratory disorders, soft-tissue disorders and digestive disorders. Together with the potential causal effects that we were not able to follow-up, these findings support a role for insomnia symptoms in multimorbidity. The findings also suggest that effective insomnia treatments, such as the cognitive behavioural therapy-insomnia [25], which has been shown to be an effective treatment for depression when comorbid with insomnia [133], could be used to treat a range of other adverse health-related outcomes; however, this requires further investigation.

We found evidence (which was directionally consistent across the MR-PheWAS, the two-sample follow-up and the two-sample sensitivity analyses, and for which the 95% CIs excluded the null in the former two) for a number of outcomes which have not been explored in MR research. These outcomes were spondylosis, unspecified joint disorders, shoulder lesions, unspecified soft-tissue disorders, gastritis (including duodenitis), oesophagitis, diverticular disease of intestine, diaphragmatic hernia, bronchitis, unspecified headache disorders and C-reactive protein levels. The bidirectional relationship between insomnia and headache has been extensively researched in previous non-MR literature [134]. Furthermore, a positive association between insomnia and C-reactive protein levels has previously been shown in standard observational research [135]. C-reactive protein is a marker of inflammation which is itself a response of the immune system, providing evidence that insomnia may affect the immune system. The relationship between insomnia and the other outcomes has not been extensively researched in conventional epidemiology studies and these are, therefore, novel findings. However, diaphragmatic hernia, is a birth defect and so it is implausible this could be caused by insomnia, indicating the results are subject to violations of the core assumptions.

### Strengths and limitations

A key strength of our hypothesis-free MR-PheWAS is that it allows for many potential novel causal effects of insomnia symptoms to be identified. Furthermore, we used two-sample MR to follow up as many of the potential causal effects as possible and included sensitivity analyses to explore potential bias due to horizontal pleiotropy.

Limitations include variations in power due to the differing numbers of samples and cases across UK Biobank phenotypes meaning our MR-PheWAS analyses may have been underpowered for some outcomes. For the two-sample MR analyses, sample sizes ranged between 1000 and 360,838 for the outcome GWASs. With larger sample sizes, more precise estimates may have been obtained. Also, 366 (84%) potential causal effects could not be followed up because we were unable to identify suitable summary GWAS data in MR-Base. It is possible that for some outcomes, suitable GWASs may exist but may not have been added to MR-Base or may have become available after the search was conducted. As GWASs are conducted for a wider range of outcomes and GWASs increase in size, future research should explore avenues not currently explored in our follow-up and update the current analyses to increase power. We did update all FinnGen GWASs to the most recent versions which were released after the search for GWASs and screening was completed, but did not search for new GWASs specifically. In the two-sample MR follow-up, there was overlap between a number of the outcome GWASs and the exposure GWAS. This has the potential to bias the results away from the null; however, previous research has suggested sample overlap often does not have a large effect [136].

It is possible that some of the potential causal effects of insomnia that we have identified are driven by the health outcome in question causally influencing insomnia [33]. As GWASs get larger, they are more likely to identify genome-wide significant associations for phenotypes that are downstream of other health-related factors. For example, previous MR studies have shown that depression affects insomnia [12, 18], and a large GWAS of insomnia might identify statistically robust SNPs associated with insomnia, some of which are identified because of the relationship of depression with insomnia. Given the number of outcomes explored in this study, investigating reverse causality is left to future work. It is also possible that the results are subject to horizontal pleiotropy. In our two-sample follow-up, we used sensitivity analyses to explore bias due to unbalanced horizontal pleiotropy. These methods do not look at specific hypothesised pleiotropic paths but rather help to see whether pleiotropic paths might have biased estimates.

The questionnaires that were used in the GWAS that provided our genetic instruments are widely used in observational studies. They reflect a person’s subjective reporting of symptoms, which may not be consistent of a diagnosis of insomnia. That said clinical diagnostic codes misclassify an important number who would meet diagnostic criteria as not everyone with symptoms will seek clinical help and not all of those who do will be diagnosed in the same way [137]. Furthermore, there may be differences in the health effects of short- and long-term insomnia and the insomnia definition used in the GWAS does not acknowledge the length of time the symptoms have been experienced, only whether they are present or not. Also, the non-representativeness of UK Biobank may also bias the results. Finally, it is important to note our presentation of MR-PheWAS results as proportions of potential causal effects in different phenotypic categories, which, although a useful summary, may be misleading if the correlations within each category differs across categories.

## Conclusions

Our results suggest that insomnia symptoms may have broad effects on health. In particular, we identified novel effects (that replicated in follow-up analyses) on respiratory disorders, soft-tissue disorders and digestive disorders and confirmed previously identified effects on mental health, hyperglycaemia, pain and body composition outcomes. These findings support a role for insomnia symptoms in multimorbidity and the possibility that effective insomnia treatments should be integrated into the treatment of other diseases. Future research should follow up individual outcomes in greater depth, including novel methods being developed for time-varying exposures and non-linear associations, to confirm novel findings.

## Supplementary Information

**Additional file 1:** **Text S1.** UK Biobank Sample. **Text S2.** Insomnia Phenotype. **Text S3.** Sensitivity Analyses Methods. **Text S4.** Sensitivity Analysis Results. **Figure S1.** Flow chart of participant inclusion. **Figure S2.** Odds ratio and 95% confidence interval for association between each SNP used in the main GRS and insomnia in UK Biobank (Field 1200, with an answer of “usually” coded as an insomnia case). **Figure S3.** Odds ratio and 95% confidence interval for association between each SNP used in the S1 and S2 GRS and insomnia in UK Biobank (Field 1200, with an answer of “usually” coded as an insomnia case). **Figure S4.** Venn diagram of the number of GRS-outcome associations which passed the Bonferroni-corrected significance threshold for each MR-PheWAS (the percentages are with respect to the total number of associations (542) identified across all MR-pheWAS). **Figure S5.** Flow chart of GWAS inclusion for follow-up. **Figure S6.** Prisma style flow chart for article screening in systematic search.

**Additional file 2:** **Table S1.** GWAS significant SNPs in 23&Me used to construct GRS for PheWAS and used to conduct two-sample MR follow-up. *rs28780988 used as a proxy for rs28458909 which was identified as an independent GWAs significant SNP in the clumping of the 23andMe GWAS results but was not available in UK biobank. **Table S2.** SNPs which were GWAS significant in both UK Biobank/23andMe meta-analysis and 23&Me, used to construct GRS for sensitivity PheWAS analyses. **Table S3.** Results from the MR-PheWAS and sensitivity analysis 1 and 2 for each outcome (Ordered by the p-value from the main MR-PheWAS). For linear regressions the beta is the mean difference per one standard deviation increase in GRS and for all others the beta is the odds ratio per one standard deviation increase in GRS. **Table S4.** Quantified details of the total number in each category, number and percentage of outcomes reaching criteria for potential causal effect in each category. **Table S5.** Quantified details of the total number in each subcategory, number and percentage of outcomes reaching criteria for potential causal effect in each subcategory. **Table S6.** Follow-up information for associations with Insomnia that passed the Bonferroni-corrected significance threshold in the main MR-PheWAS. **Table S7.** List of GWAS from TwoSampleMR package v0.5.6 included in follow-up. The potential causal effects from the MR-PheWAS these relate to are in the Outcome column separated by semicolons. **Table S8.** List of GWAS from TwoSampleMR package v0.5.6 returned in search but not included in follow-up. The Reason column gives the reason for exclusion. **Table S9.** Results from two-sample MR follow-up for binary outcomes. **Table S10.** Results from two-sample MR follow-up for continuous outcomes. **Table S11.** Articles identified in systematic search and included after screening, with a summary of findings. **Table S12.** Articles identified in systematic search and excluded at full text screening.

## Data Availability

All data is available on request from the UK Biobank or 23andMe. The full GWAS summary statistics for the 23andMe discovery data set will be made available through 23andMe to qualified researchers under an agreement with 23andMe that protects the privacy of the 23andMe participants. Please visit https://research.23andme.com/collaborate/#dataset-access/ for more information and to apply to access the data. All code used in the analyses is available at https://github.com/MRCIEU/PHESANT-MR-PheWAS-Insomnia.

## Abbreviations

CI: Confidence interval
GRS: Genetic risk score
GWAS: Genome-wide association study
IVW: Inverse-variance weighted
LD: Linkage disequilibrium
MR: Mendelian randomisation
OR: Odds ratio
PheWAS: Phenome-wide association study
SD: Standard deviation
SNP: Single-nucleotide polymorphism
