# Supplementary material for: Identifying the potential causal role of insomnia symptoms on 11,409 health-related outcomes: a phenome-wide Mendelian randomisation analysis in UK Biobank
Source: BMC Med. 2023 Apr 3;21:128. doi: 10.1186/s12916-023-02832-8 (PMC10071698; doi:10.1186/s12916-023-02832-8)
Supplement: Supplementary file 1 — Additional file 1: Text S1. UK Biobank Sample. Text S2. Insomnia Phenotype. Text S3. Sensitivity Analyses Methods. Text S4. Sensitivity Analysis Results. Figure S1. Flow chart of participant inclusion. Figure S2. Odds ratio and 95% confidence interval for association between each SNP used in the main GRS and insomnia in UK Biobank (Field 1200, with an answer of “usually” coded as an insomnia case). Figure S3. Odds ratio and 95% confidence interval for association between each SNP used in the S1 and S2 GRS and insomnia in UK Biobank (Field 1200, with an answer of “usually” coded as an insomnia case). Figure S4. Venn diagram of the number of GRS-outcome associations which passed the Bonferroni-corrected significance threshold for each MR-PheWAS (the percentages are with respect to the total number of associations (542) identified across all MR-pheWAS). Figure S5. Flow chart of GWAS inclusion for follow-up. Figure S6. Prisma style flow chart for article screening in systematic search. [file 12916_2023_2832_MOESM1_ESM.docx]

**Identifying the potential role of insomnia on multimorbidity: A Mendelian randomization phenome-wide association study in UK Biobank**

Mark Gibson et al.

**Supplementary Text and Figures**

**Supplementary Text**

**Methods**

*S1 -* *UK Biobank Sample*

UK Biobank genetic data version 3 (2018) was used. The UK BiLEVE Axiom array (n=50,520) and UKB Axiom array (n=438,692), were used for genotyping and covered 812,428 genetic markers. Of these 805,426 markers passed genotype quality controls. Imputation was conducted using the Haplotype Reference Consortium reference panel16 (version 1.1), and a reference panel made from the combination of UK10K and the 1000 Genomes project. Imputed data was available for 92,693,895 variants.

*S2 -* *Insomnia Phenotype*

Insomnia cases were identified in UK Biobank (Field 1200) by the participant answering “Usually” to the question “Do you have trouble falling asleep at night or do you wake up in the middle of the night?” as part of the touchscreen questionnaire with participants answering “usually” rather than “never/rarely” or “sometimes” being identified as cases. Participants could also answer with “I do not know” and “Prefer not to answer”. Insomnia cases made up 28% of our final UK Biobank sample.

Insomnia cases were identified in 23andMe by the participant answering yes (or selecting any of the relevant multiple choice answers) to one of the following questions:

Have you ever been diagnosed with, or treated for: Insomnia?

Have you ever been diagnosed with, or treated for, any of the following conditions?: Insomnia but not Narcolepsy, Sleep apnea or Restless leg syndrome.

Has a doctor ever told you that you have any of these conditions?: Insomnia (difficulty getting to sleep or staying asleep).

Have you ever been diagnosed by a doctor with any of the following neurological conditions?: Sleep disturbance.

Do you routinely have trouble getting to sleep at night?

What sleep disorders have you been diagnosed with? Please select all that apply: Insomnia, trouble falling or staying asleep.

Have you ever taken these medications?: Prescription sleep aids.

In the last 2 years, have you taken any of these medications?: Prescription sleep aids.

Controls were identified by participants answering no to all of the following questions:

Have you ever been diagnosed with, or treated for Insomnia, Narcolepsy, Sleep apnea, Restless leg syndrome?

Have you ever been diagnosed with or treated for any of the following conditions?: Post-traumatic stress disorder (PTSD), Autism, Asperger's, Sleep disorder.

Have you ever been diagnosed with or treated for a sleep disorder?

*S3 -* *Sensitivity Analyses Methods*

These GRSs included 111 SNPs (Supplementary Table S2) identified in a meta-analysis GWAS of both 23andMe and UK Biobank. The first sensitivity GRS (S1) weighted SNPs by the per-allele association with insomnia symptoms from the pooled UK Biobank and 23andMe analyses. The second sensitivity GRS (S2) weighted by the 23andMe per allele associations with insomnia symptoms. While 114 independent SNPs were identified in the original GWAS 3 SNPs were removed for the following reasons. One SNP (rs9540729) was palindromic and had an EAF in 23andMe between 0.49 and 0.51 meaning it could not be aligned with the UK Biobank data. Therefore, it was excluded from both scores for consistency. Two SNPs, rs77641763 and rs117630493, had point estimates in the meta-analysis GWAS which were in the opposite direction to the 23andMe GWAS, and so were also removed. Of the SNPs included in the score, 38 were also used in the main GRS. The authors of the original GWAS used UK Biobank data to calculate LD and an LD threshold of R^2^>0.001 was used to define independent SNPs.

**Results**

*S4 -* *Sensitivity Analysis Results*

The two GRS used in sensitivity analyses were also associated with an increased risk of insomnia symptoms in UK Biobank: OR=1.11 [95%CI: 1.10, 1.12] per one standard deviation increase in the S1 GRS (*p*=1.65x10^-157^, McFadden’s pseudo R^2^=0.01); OR=1.10 [95% CI: 1.10, 1.11] per one standard deviation increase in the S2 GRS (p=6.76x10^-145^, McFadden’s pseudo R^2^=0.01). The correlations between each score were strong (r=0.99 [95% CI: 0.99, 0.99] for the S1 GRS with the S2 GRS; r=0.69 [95% CI: 0.69, 0.69] for the S1 GRS with the main GRS; r=0.70 [95% CI: 0.69, 0.70] for the S2 GRS with the main GRS). See Supplementary Figure S3 for associations between each SNP and insomnia symptoms for the S1 and S2 GRS.

For GRS S1 and S2, 498 and 490 associations were identified as potential causal effects respectively. There was considerable concordance between the three GRS, with 72% of the 542 associations identified using at least one GRS identified using all three. For associations identified as potential causal effects in any of the three GRS, the association was directionally consistent across all three.

**Supplementary Figures**

**
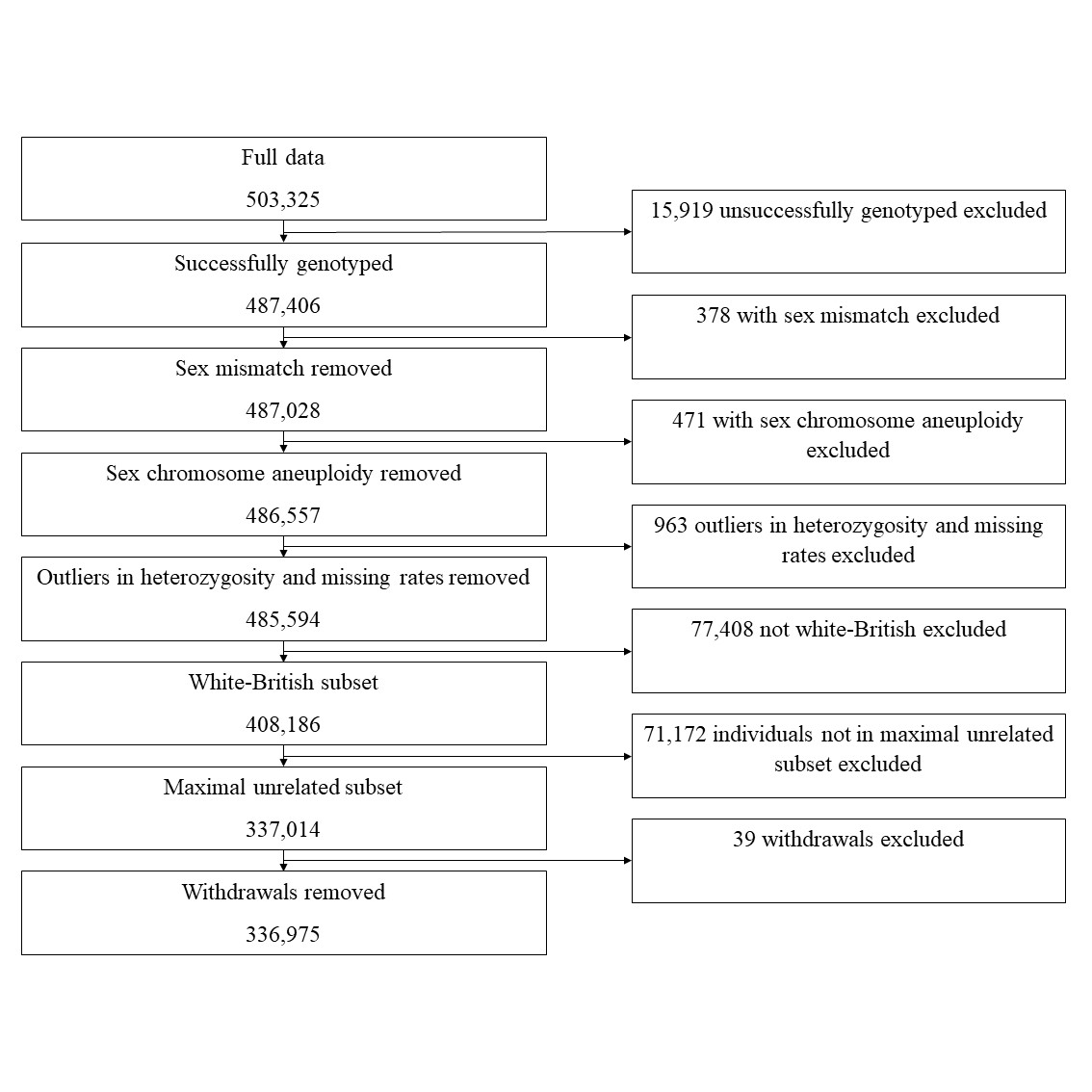
**

**Figure S1:** Flow chart of participant inclusion.

**
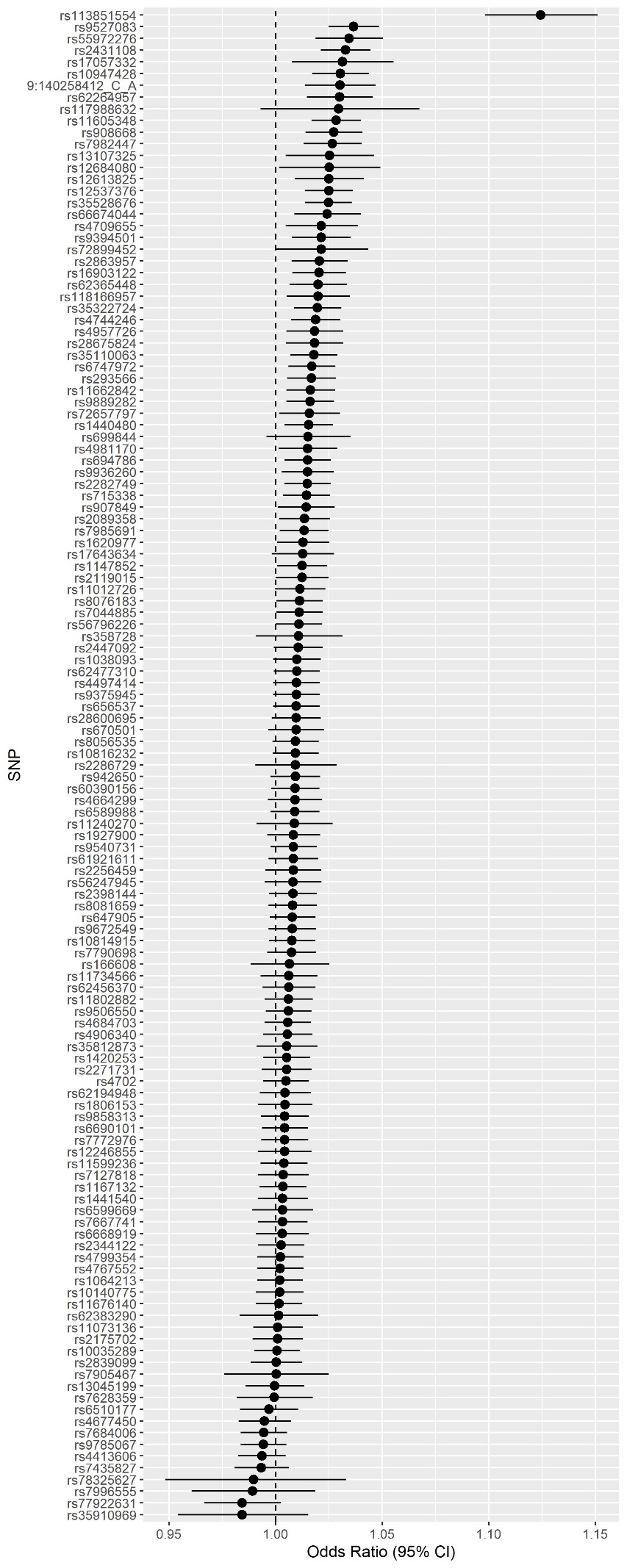
Figure S2:** Odds ratio and 95% confidence interval for association between each SNP used in the main GRS and insomnia in UK Biobank (Field 1200, with an answer of “usually” coded as an insomnia case).

**
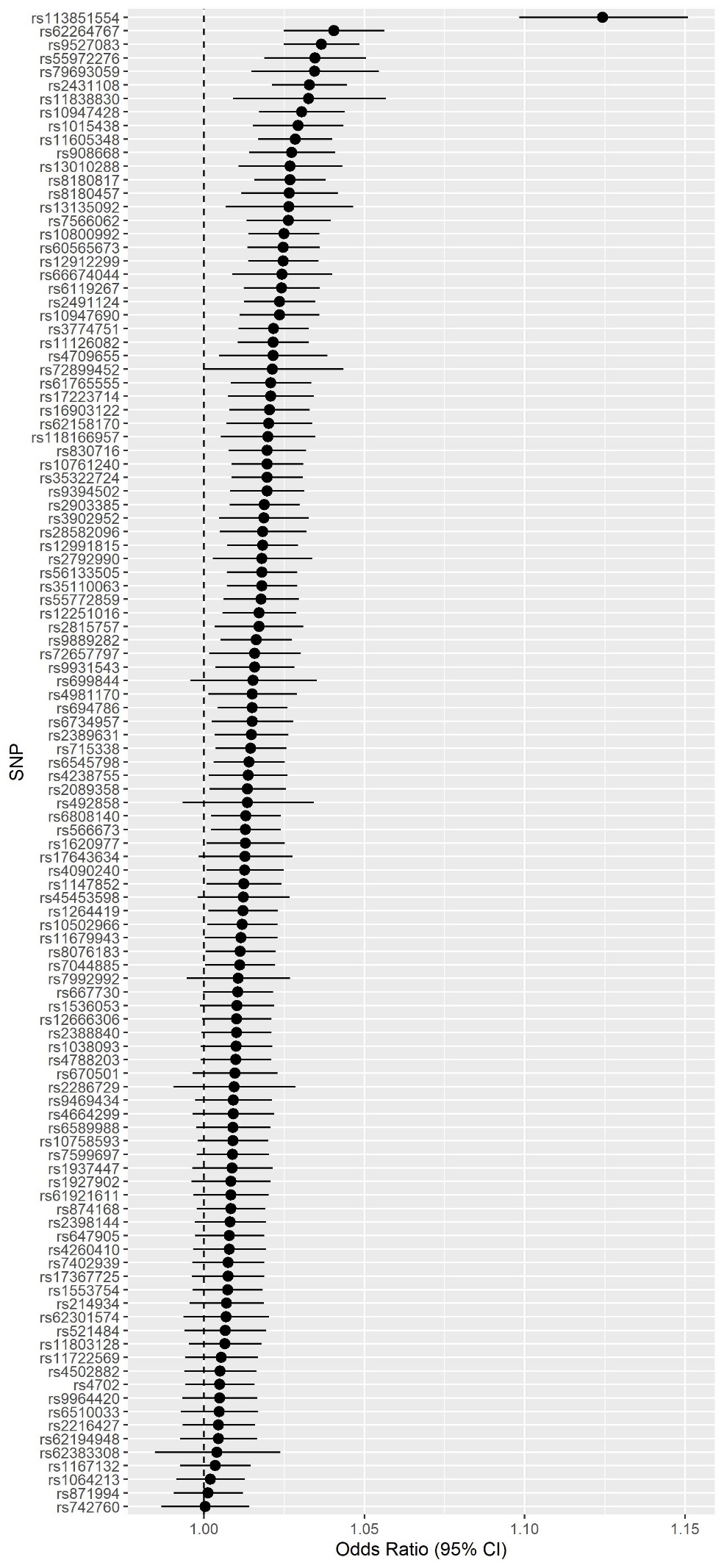
**

**Figure S3:** Odds ratio and 95% confidence interval for association between each SNP used in the S1 and S2 GRS and insomnia in UK Biobank (Field 1200, with an answer of “usually” coded as an insomnia case).

**
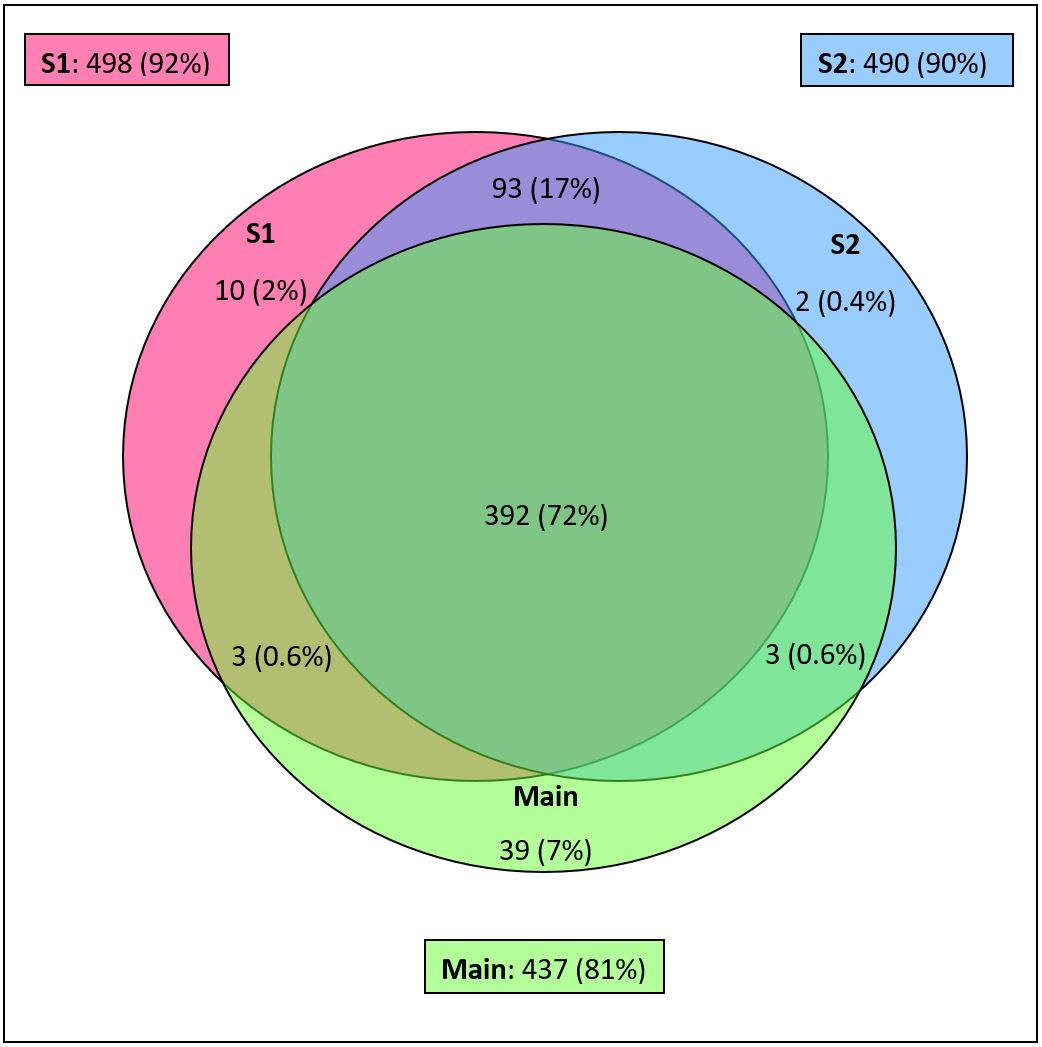
 Figure S4:** Venn diagram of the number of GRS-outcome associations which passed the Bonferroni-corrected significance threshold for each MR-PheWAS (the percentages are with respect to the total number of associations (542) identified across all MR-pheWAS).

**
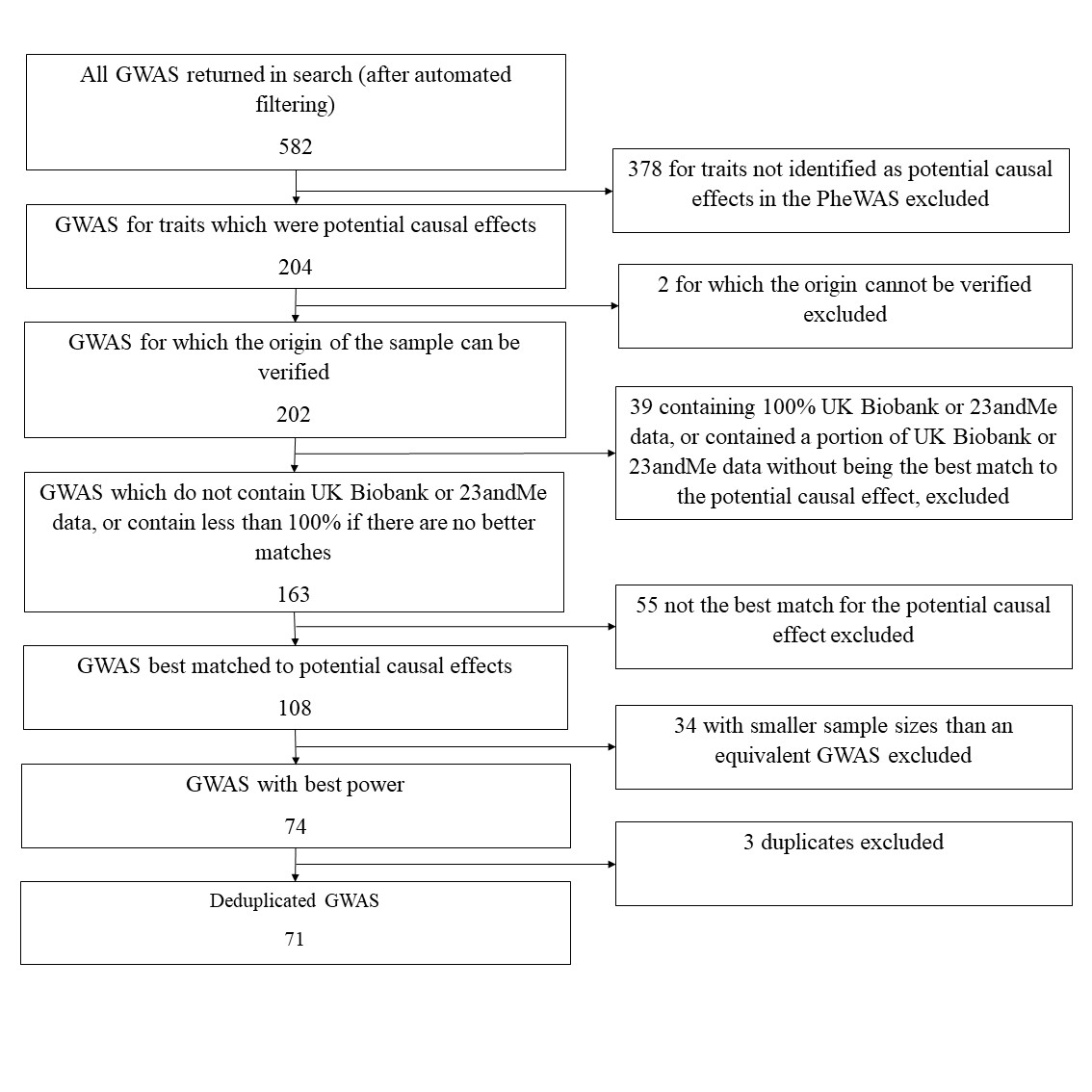
 Figure S5:** Flow chart of GWAS inclusion for follow-up.

**
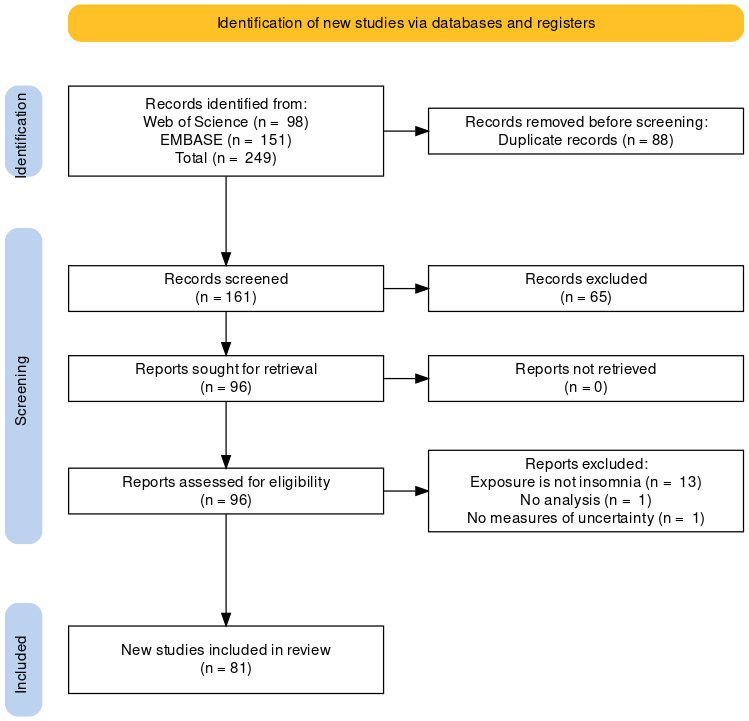
 Figure S6:** Prisma style flow chart for article screening in systematic search.
